# ***ASPERGILLUS LUCHUENSIS*** **, AN INDUSTRIALLY IMPORTANT BLACK** ***ASPERGILLUS*** **IN EAST ASIA**

**DOI:** 10.1371/journal.pone.0063769

**Published:** 2013-05-28

**Authors:** Seung-Beom Hong, Mina Lee, Dae-Ho Kim, Janos Varga, Jens C. Frisvad, Giancarlo Perrone, Katsuya Gomi, Osamu Yamada, Masayuki Machida, Jos Houbraken, Robert A. Samson

**Affiliations:** 1 Korean Agricultural Culture Collection, Agricultural Microbiology Division, National Academy of Agricultural Science, RDA, Suwon, Korea; 2 Department of Microbiology, Faculty of Science and Informatics, University of Szeged, Szeged, Hungary; 3 Center for Microbial Biotechnology, Department of Systems Biology, Technical University of Denmark, Lyngby, Denmark; 4 Institute of Sciences of Food Production, CNR, Via Amendola, Bari, Italy; 5 Laboratory of Bioindustrial Genomics, Department of Bioindustrial Informatics and Genomics, Graduate School of Agricultural Science, Tohoku University, Aoba-ku, Sendai, Japan; 6 Department National Research Institute of Brewing, Higashi-Hiroshima, Japan; 7 Bioproduction Research Institute, National Institute of Advanced Industrial Science and Technology (AIST), Higashi 1-1-1, Ibaraki, Japan; 8 Genome Biotechnology Laboratory, Kanazawa Institute of Technology, Yatsukaho 3-1, Hakusan, Ishikawa, Japan; 9 CBS-KNAW Fungal Biodiversity Centre, Utrecht, the Netherlands; University of Missouri, United States of America

## Abstract

Aspergilli known as black- and white-koji molds which are used for awamori, shochu, makgeolli and other food and beverage fermentations, are reported in the literature as *A. luchuensis, A. awamori, A. kawachii,* or *A. acidus.* In order to elucidate the taxonomic position of these species, available ex-type cultures were compared based on morphology and molecular characters. *A. luchuensis*, *A. kawachii* and *A. acidus* showed the same banding patterns in RAPD, and the three species had the same rDNA-ITS, β-tubulin and calmodulin sequences and these differed from those of the closely related *A. niger* and *A. tubingensis*. Morphologically, the three species are not significantly different from each other or from *A. niger* and *A. tubingensis*. It is concluded that *A. luchuensis, A. kawachii* and *A. acidus* are the same species, and *A. luchuensis* is selected as the correct name based on priority. Strains of *A. awamori* which are stored in National Research Institute of Brewing in Japan, represent *A. niger* (n = 14) and *A. luchuensis* (n = 6). The neotype of *A. awamori* (CBS 557.65 =  NRRL 4948) does not originate from awamori fermentation and it is shown to be identical with the unknown taxon *Aspergillus welwitschiae*. Extrolite analysis of strains of *A. luchuensis* showed that they do not produce mycotoxins and therefore can be considered safe for food and beverage fermentations. *A. luchuensis* is also frequently isolated from meju and nuruk in Korea and Puerh tea in China and the species is probably common in the fermentation environment of East Asia. A re-description of *A. luchuensis* is provided because the incomplete data in the original literature.

## Introduction

Several species belonging to *Aspergillus* section *Nigri* are associated with food fermentations in East Asia. For example, *A. luchuensis* and *A. awamori* (black-koji molds) are linked with the production of awamori, a distilled alcoholic beverage made on Okinawa island in Japan, *A. kawachii* and *A. coreanus* (white-koji molds) with the making of shochu and makgeolli [1], [2], [3], [4], [5], [6]. The black and white-koji molds are used to make koji (moldy material) for awamori, shochu and makgeolli fermentations, which provides various enzymes for maceration and saccharification of raw materials such as rice, barley, and sweet potatoes, as well as a large amount of citric acid for maintaining the fermentation mash at low pH to prevent from contamination of wild microorganisms.

The taxonomy of section *Nigri* is revised various times [7], [8], [9] and western taxonomists have accepted other species in this section than mycologists in East Asia. For example, Sakaguchi *et al.* [10] and Murakami [8] accepted *A. luchuensis*, and recently, Yamada *et al.* [11] proposed that industrial black-koji molds, including *A. kawachii* (an albino mutant of black-koji mold) be named *A. luchuensis*. In contrast, western mycologists have not accepted *A. luchuensis* because there has been confusion about the validity of the name. Inui [1], [2] described *A. luchuensis* as a mold used for the production of awamori on the Okinawa islands of Japan, but it is considered doubtful because it was described with uniseriate conidial heads, while biseriate heads can be observed in the ex-type culture. Samson *et al.* [12] and Varga *et al.* [9] re-described *A. acidus* for strains used for awamori fermentations. This name is based *A. aureus* var. *acidus* Nakazawa *et al.*, isolated from awamori-koji in Okinawa in 1936. Mogensen *et al.* [13] reported that *A. acidus* is the dominant microorganism in the Puerh tea which is a variety of post-fermented tea produced in Yunnan province in China.

Raper and Fennell [7] used CBS 557.65 ( =  NRRL 4948), which does not originate from awamori Koji, for their description of *A. awamori*, and subsequently Al-Musallam [14] designated the strain as neotype of *A. niger* var. *awamori*. Based on this neotype, Perrone *et al.* [15] re-established *A. awamori* as a phylospecies in *Aspergillus* section *Nigri* based on multigene sequencing of isolates found on grapes. However, the name, *A. awamori* implies that this species is associated with black koji fermentations and awamori production. In fact, Perrone *et al.* [15] demonstrated in his study that the *A. awamori* strains used in the Japanese koji fermentation do not belong to his proposed *A. awamori* phylospecies.

The taxonomic position of species used in black- and white-koji fermentations is re-investigated. We studied the original descriptions of *A. acidus*, *A. kawachii*, *A. luchuensis* and *A. coreanus* and compared the available ex-type strains of these species with other species belonging to section *Nigri* using ITS, β-tubulin and calmodulin gene sequences and RAPD profiles. Furthermore, the taxonomic position of *Aspergillus awamori* was re-investigated.

## Materials and Methods

Ex-types of *A. luchuensis, A. acidus* and *A. kawachii* were obtained from NBRC (NITE Biological Resources Center, Japan) and compared with cultures deposited at CBS (CBS-KNAW Fungal Biodiversity Centre, the Netherlands), KACC (Korean Agricultural Culture Collection, Korea) and IBT (Center for Microbial Biotechnology, Denmark). A strain of *A. kawachii* (KACC 46516) which is used for rice-koji for making makgeolli in Korea, and the ex-type strain of *A. coreanus* (KACC 41731^T^) were obtained from the Chungmu company (Korea, Ulsan) and Dr Tae Shick Yu (Keimyung University, Taegu, Korea) respectively. Additionally, two *Aspergillus* strains were isolated from traditional nuruk (KACC 46420) and meju (KACC 46490) in Korea. Detailed information of the strains is given in Table 1.

**Table 1:** Fungal strains used in this study.

KACC No.	Other Collection no.	Species & type information	Origin and information	ITS GenBank no.	β-tubulinGenBank no.	Calmodulin GenBank no.
46772	NBRC* 4281, RIB* 2642,IFM* 47726, CBS* 205.80	Extype of *A. luchuensis*	Awamori-koji, Okinawa, Japan.	JX500081	JX500062	JX500071
46771	NBRC 4308	Extype of *A. kawachii*	Shochu-koji, Kyusyu, Japan	JX500082	JX500063	JX500072
46516	CF* 1005	*A. kawachii*	Makgeolli-Koji, Chungmu Fermentation co., Korea	JX500083	JX500064	JX500073
45131	CBS 564.65, ATCC* 16874, NBRC 4121, IMI* 104688, NRRL* 4796	Extype of *A. acidus*	Awamori-koji, Okinawa, Japan.	JX500084	GU296697	JX500074
45132	CBS 106.47	*Aspergillus* sp.	Switzerland.	JX500085	JX500065	JX500075
45133	CBS 124.49	*Aspergillus* sp.	Central America	JX500086	JX500066	JX500076
41731	CBS 119384	*A. acidus* (extype of *A. coreanus*)	Nuruk, Korea	JX500087	JX500067	JX500077
46420		*Aspergillus* sp.	Nuruk, Korea	JX500088	JX500068	JX500078
46490		*Aspergillus* sp.	Meju, Korea	JX500089	JX500069	JX500079
	CBS 139.54	Epitype of *A. welwitschiae*	female inflorescence of *Welwitschia mirabilis*collected in Namibia	FJ629340	FJ629291	KC480196
45072	CBS 554.65, ATCC 16888, NBRC 33023, IMI 050566, NRRL 326	Type of *A. niger*	Tannin-gallic acid fermentation, Connecticut, USA	JX500090	JX500070	JX500080
46805	CBS 134. 48	Type of A. *tubingensis*	R. Mosseray, No. 726	AJ223853	AY820007	AJ964876

* ATCC, American Type Culture Collection, USA; CBS, CBS-KNAW Fungal Biodiversity Centre, The Netherlands; CF, Chungmu Fermentation Co., Korea; IFM, Institute for Food Microbiology (at present, the Research Center for Pathogenic Fungi and Microbial Toxicoses), Japan; IMI, CABI Culture Collection, UK; KACC, Korean Agricultural Culture Collection, Korea; NBRC, NITE Biological Resources Center, Japan; NRRL, ARS Culture Collection, USA; RIB, National Research Institute of Brewing, Japan.

The morphological characters were examined by the methods described in Varga *et al.* [2011]. In addition, the ex-type culture of *A. luchuensis* was grown on steamed rice for microscopy to compare the observations by Inui [1], [2] who used this medium to describe the species. The RAPDs in this study were performed according to Hong *et al.* [16]. In order to determine phylogenetic relationship of the strains, partial fragments of the β-tubulin, calmodulin and ITS were sequenced by the methods of Peterson [17] and combined. For comparing with the other species in *Aspergillus* section *Nigri,* sequences were obtained from Varga *et al.* [9] and combined. Combined DNA data were analyzed using Tamura-Nei parameter distance calculation model, which was then used to construct the Neighbor-Joining (NJ) tree with MEGA version 5 [18]. Newly generated sequences were deposited in GenBank under accession numbers JX500062 - JX50090 (Table 1).

Sequence comparison between *A. awamori sensu* Perrone *et al.* [15], *A. welwitschiae* and *A. niger* strains was conducted on the basis of beta-tubulin, calmodulin, translation elongation factor-1 alpha data from Perrone *et al.* [15], and by adding RNA polymerase II sequences amplified using primers 5F and 7CR [19]. The analysis was conducted on a total of 100 strains grouped in *A. awamori* clade and 20 strains grouped as *A. niger* [Perrone, unpublished data]. The position of the sequence difference were fixed by comparison to the complete sequences of each gene recovered from *A. niger* CBS 513.88 genome; for calmodulin gene the NCBI ID = NT_166539, for β-tubulin NCBI ID = AM270165.1, for translation elongation factor-1 NCBI ID = NT166533 and for RPB2 NCBI ID = XM_001395124.2.

For the extrolite analysis cultures were grown on the agar media CYA and YES for 7 days at 25°C prior to extraction. Extrolites were analyzed by HPLC using alkylphenone retention indices and diode array UV-VIS detection as described by Houbraken *et al.* [20].

## Results

**Figure 1:**
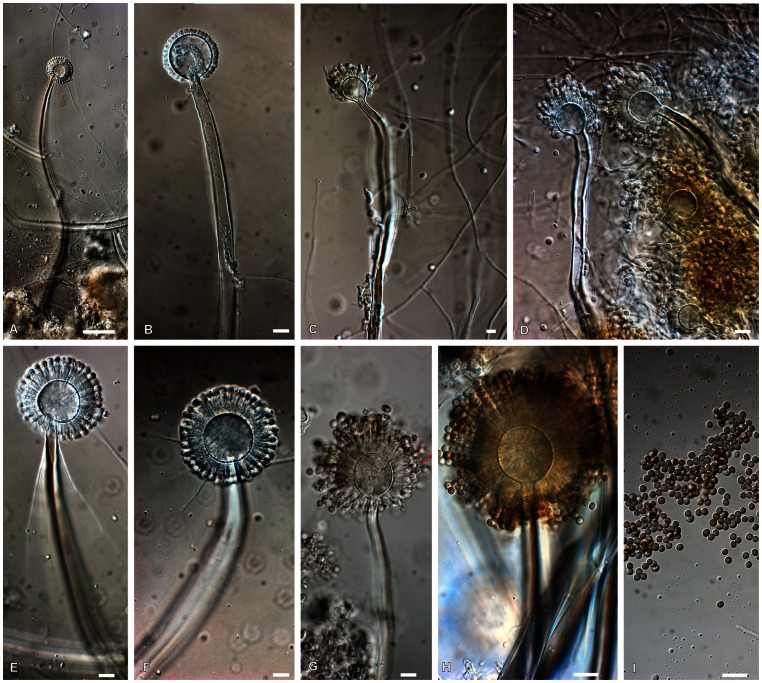
A–I. Conidiophore structures and conidia of NBRC 4281, the ex-type of *A.luchuensis* grown for 3 days on steamed rice. Scale bars in all figure = 10 µm, except A = 100 µm.

The morphology on agar media of the ex-type of *A. luchuensis* (KACC 46772^ T^ = NBRC 4281^ T^), *A. kawachii* (KACC 46771^T^ = NBRC 4308^T^, KACC 46516) and *A. acidus* (KACC 45131^T^, KACC 41731) proved to be similar (Table S1, Figure S1), although the colony colors varied. The above five strains have shorter conidiophores and less roughened conidia than *A. niger* (KACC 45072^T^) and *A. tubingensis* (KACC 46805^T^). All strains including the ex-type of *A. luchuensis* have biseriate conidial heads on Malt Extract Agar (Figure S1), although aberrant conidial heads could be observed. No morphological differences were observed between the ex-types of *A. luchuensis* and *A. acidus* (Table S1). A detailed morphological study of the ex-type strain of *A. luchuensis* grown on steamed and sterilized rice for three days at 25°C, showed that the conidial heads were often uniseriate (Fig. 1). These conidial heads structures strongly resemble those illustrated by Inui [1], [2]. After five days, the conidial heads were more frequently biseriate although irregular formation of metulae could be observed.

**Figure 2:**
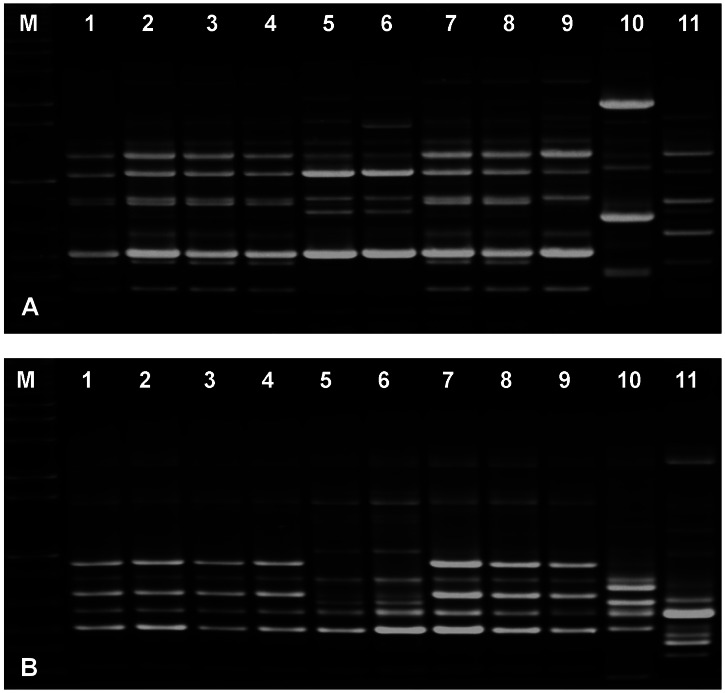
Comparison of RAPD patterns among *Aspergillus luchuensis*, *A. acidus* and *A. kawachii*. **Primers PELF (A) and URP1F (B) were used for RAPDs.** Lane M, Size marker; 1, Extype of *A. luchuensis* (KACC 46772); 2, Extype of *A. kawachii* (KACC 46771); 3, *A. kawachii* (KACC 46516); 4, Extype of *A. acidus* (KACC 45131); 7, *A. acidus* (KACC 41731), 4–5 and 7–8, *Aspergillus* sp. (KACC 45132, 45133, 46420, 46490, respectively); 10, Type of *A. niger* (KACC 45072); 11, Type of *A. tubingensis* (KACC46805).

In our RAPD experiments with the primers PELF and URP1F, the ex-type strain of *A. luchuensis* (KACC 46772^ T^), two strains of *A. kawachii* (KACC 46771^ T^ and 46516), two strains of *A. acidus* (KACC 45131^ T^, 41731) and two strains of an *Aspergillus* sp. (KACC 46420, 46490) showed identical band patterns (Fig. 2). The band patterns of them were similar to those of two strains of *Aspergillus* sp. (KACC 45132, 45133), but were different from those of *A. niger* (KACC 45072^T^) and *A. tubingensis* (KACC 46805^T^).

**Figure 3:**
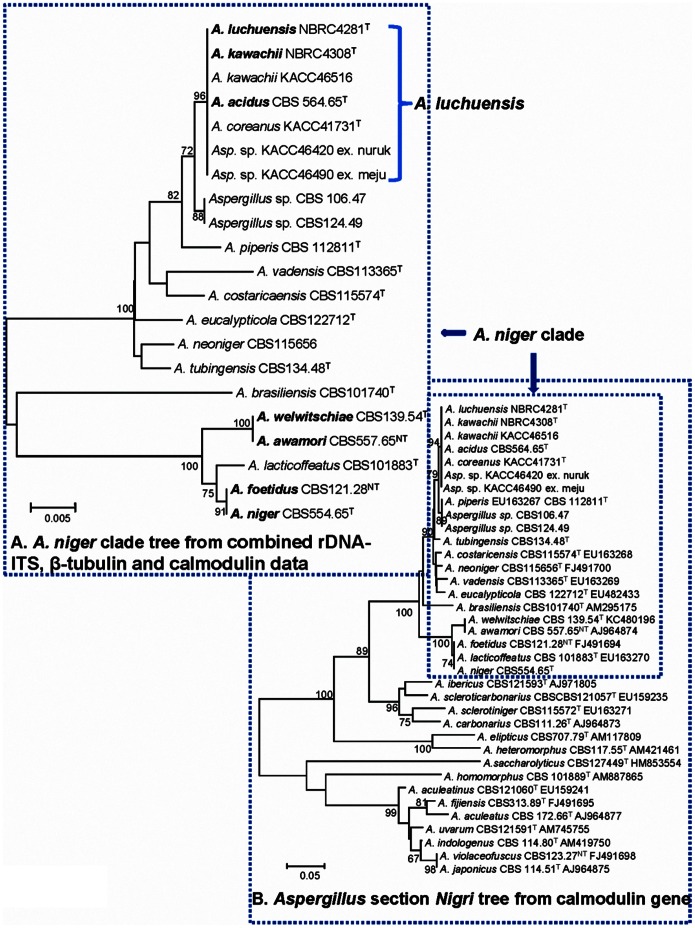
Phylogenetic tree of *Aspergillus luchuensis*, *A. acidus*, *A. kawachii*, *A. welwitschiae* and related species in *A. niger* clade inferred from Neighbor-joining analysis of combined rDNA-ITS, β-tubulin and calmodulin data (A) and tree of *Aspergillus* section *Nigri* from partial calmodulin gene (B). Bootstrap analysis was performed with 1,000 replications.

The combined analysis of β-tubulin, calmodulin and ITS sequences show that the strains of *A. luchuensis*, *A. kawachii*, *A. acidus* and *Aspergillus* sp. have 100% sequence homology and they are positioned distantly from *A. tubingensis* and *A. niger* (Fig. 3). The neotype of *A. awamori* CBS 557.65^NT^ is on well-supported branch (100% bootstrap value) with the type strain of *A. niger* CBS 554.65^T^.

*A. awamori* strains isolated from oriental food fermentation process could be accommodated in four groups. Of 38 *A. awamori* strains, 24 strains clustered into *A. niger* group, 10 into *A. luchuensis* group and two into *A. tubingensis.* Two isolates could be classified as *A. awamori sensu* Perrone *et al.* (Table 2). These two strains did not originate from awamori-koji.

**Table 2:** Species assignment of isolates deposited in the National Research Institute Brewing (NRIB), CBS and IBT collections, mostly isolated from awamori or other oriental food fermentation processes.

Isolate no.	Other Collection no.	Name as deposited	origin	Identification based on β-tubulin sequence
CBS 101701	IBT 19347 = IFO 8877	*A. awamori*	J. Sugiyama	*A. niger*
CBS 111.34		*A. awamori*	R. Nakazawa	*A. luchuensis*
CBS 112.52	NRRL 4840	*A. aureus* var. *acidus*	R. Ciferri	*A. niger*
CBS 113.33	NRRL 4864	*A. niger* mut. *fusca*	A. Blochwitz	*A. niger*
CBS 113.52	NRRL 4841	*A. aureus* var. *brevior*	R. Ciferri	*A. niger*
CBS 115.52	ATCC 11358	*A. awamori*	K. Sakaguchi	*A. luchuensis*
CBS 115988	NRRL 3112	*A. awamori*		*A. niger*
CBS 117.51	NRRL 4859	*A. miyakonensis*	K. Kominami	*A. niger*
CBS 117.52	NRRL 4844	*A. awamori* var. *fuscus*	R. Ciferri	*A. niger*
CBS 118.35	NRRL 4883	*A. hennebergii*	A. Blochwitz	*A. niger*
CBS 118.52		*A. awamori* var. *minimus*	R. Ciferri	*A. niger*
CBS 119.52	NRRL 4846	*A. awamori* var. *piceus*	R. Ciferri	*A. luchuensis*
CBS 121.48	NRRL 4857	*A. longobasidia*	R. Mosseray	*A. awamori/welwitschiae*
CBS 125.52	IAM 2255, JCM 22302	*A. inuii* (ex-type)	K. Sakaguchi,	*A. luchuensis*
CBS 126.52	NRRL 4860	*A. miyakonensis*	R. Ciferri	*A. tubingensis*
CBS 127.48	NRRL 4869	*A. pseudocitricus*	R. Mosseray	*A. niger*
CBS 128.48	NRRL 4870	*A. pseudoniger*	R. Mosseray	*A. tubingensis*
CBS 557.65	NRRL 4948 = ATCC 16877 = IMI 211394	*A. awamori* (ex-neotype)	K.B. Raper	*A.awamori/welwitschiae*
RIB2016	NBRC4123 = CBS 565.65	*A. foetidus* var. *pallidus*	GRIF, R. Nakazawa	*A. luchuensis*
RIB2051	NBRC6086	*A. awamori*	Higuchi Co., from tanekoji	*A. luchuensis*
RIB2061		*A. awamori*	RIB K-2, Shinzato shuzo Co.,S. Sugama *et al.* (1975)	*A. luchuensis*
RIB2601	NBRC4033 = ATCC 38854	*A. awamori*	GRIF, R. Nakazawa, from amamori-koji	*A. luchuensis*
RIB2604	NBRC4314	*A. awamori*	HUT 2105 CLMR S. Usami	*A. luchuensis*
RIB2605	NBRC4116	*A. awamori*	GRIF, R. Nakazawa	*A. luchuensis*
NBRC6082	CBS 101700 = FAT407	*A. awamori*	FAT 407 (H. Iizuka),	*A. niger*
NBRC8875	CBS 101702 = JCM 1925	*A. awamori*	TI 49 (J. Sugiyama, GKC-2-1-1(2))	*A. niger*
NBRC8876	CBS 101704 = JCM 1926	*A). awamori*	TI 50 (J. Sugiyama, PKC-6-2-1(1)), core samples from stratigraphic drillings	*A. niger*
NBRC8877	CBS 100701 = JCM 1927	*A. awamori*	TI 52 (J. Sugiyama, GKC-15-2-1(2)), core samples from stratigraphic drillings	*A. niger*
RIB1062	NBRC4125	*A. awamori*	GRIF, R. Nakazawa	*A. niger*
RIB2013	NBRC4118 = IFM 57760	*A. awamori*	GRIF, R. Nakazawa	*A. niger*
RIB2014	NBRC4031 = CBS 121.28	*A. foetidus* (ex-type)	GRIF, R. Nakazawa, from awamori-koji	*A. niger*
RIB2015	NBRC4321		HUT CLMR S. Usami	*A. niger*
RIB2602	CBS 139.52 = NBRC4388	*A. usami* (ex-type)	GIB (Tonoike) from black-koji	*A. niger*
RIB2603	NBRC4397	*A. awamori*	Matuo Co.	*A. niger*

Most isolates of *Aspergillus luchuensis* specifically produce antafumicin and/or a partially characterized metabolite provisionally called “luchuensin”, in addition to asperazine, they often produce atromentin, funalenone, pyranonigrin A, and occasionally tensidol B (Table S2).

## Discussion

### Re-introduction of *A. luchuensis*

Inui [1], [2] described *A. luchuensis* as a major fermentation agent in awamori-koji in Okinawa. He reported that the species had blackish brown, uniseriate conidial heads and finely roughened conidia. Nakazawa [3], [4] also isolated molds from awamori koji and he found black and golden yellow cultures and named them *A. awamori* and *A. aureus,* respectively. He examined the *A. luchuensis* strain obtained from T. Inui, and reported that the strain had biseriate conidial heads and had similar morphological features as *A. awamori*. He rejected *A. luchuensis* because it was described as a uniseriate species [3], [4], [8]. However, Thom and Raper [21] accepted *A. luchuensis* and accommodated the species in the *Aspergillus luchuensis* series. They described it as an *Aspergillus* with uniseriate heads with occasionally biseriate sterigmata. In contrast, Nehira [22] considered *A. luchuensis* a synonym of *A. awamori*. Raper and Fennell [7] reported that *A. luchuensis* was accepted by Japanese workers as a biseriate species but based on the original description of *A. luchuensis* with uniseriate conidial heads they rejected this taxon and accepted *A. awamori* with NRRL 4948 ( =  CBS 557.65) as representative culture. Nevertheless, *A. luchuensis* has been used by Japanese mycologists, and Murakami [8] suggested that if *A. luchuensis* was corrected as a biseriate species, it was reasonable that *A. luchuensis* was used as the main awamori koji mold. Recently, Yamada *et al.* [11] proposed that industrial black-koji molds should be classified as *A. luchuensis*.

There has been confusion about the existence of the ex-type culture of *A. luchuensis,* but a study of the data of strain deposition in NBRC showed that Inui [1], [2] sent his strain to K. Sakaguchi at the ACTU (now ATU) collection. Our observations of this ex-type of *A. luchuensis* (NBRC 4281^T^) grown on steamed rice confirm the original description of Inui (Fig. 1). In our opinion, Inui [1], [2] described the species at a young stage. This can be seen from his illustrations, where young, not fully developed conidiophores were depicted. Secondly, Inui [1], [2], in the description in the Japanese article, mentioned the single seriation of the conidial head, but he did not place importance to this feature. In his German article he did not mention the seriate structure of the conidial head either. An indication that the conidial heads could be biseriate was, that Inui [1], [2] described that the phialides (sterigmata) were longer than other *Aspergillus* species. Based on the data mentioned above, *A. luchuensis* should be accepted as a valid species.

*Aspergillus kawachii,* a white-koji mold that has been used widely in shochu making on the Kyusyu in Japan, which was probably formed by the mutation of a certain black *Aspergillus* species, is considered similar with *A. luchuensis* [5], [23]. Brewers have used the name *A. kawachii*, but Japanese mycologists have used the name, *A. luchuensis* mut. *kawachii* [23], [24], [25], because *A. kawachii* was not validly published. Yamada *et al.* [11] proposed that *A. kawachii* should be classified as *A. luchuensis* based on their multilocus sequence typing of ITS, D1D2 of LSU, histon3, β-tubulin and cytochrome b sequence data. Our results of RAPDs and MLST of ITS, β-tubulin and calmodulin also support the findings of Kitahara and Kurushima [23] and Yamada *et al.* [11]. Like *A. luchuensis*, also *A. kawachii* was described with uniseriate conidial heads [23]. In our opinion, the same situation and observations occurred here as with the description of *A. luchuensis*. In conclusion, we propose to place *A. kawachii* in synonymy with *A. luchuensis*.

Nakazawa *et al.* [26] isolated black-koji molds from various awamori fermentations and proposed *A. awamori* with five varieties, *A. aureus* with five varieties and *A. miyakoensis*. One of the five varieties of *A. aureus* is variety *acidus*, which was renamed as *A. foetidus* var. *acidus* by Thom and Raper [21 Later the variety was raised to species level by Kozakiewicz [27] based on morphological features including the conidial ornamentation observed by scanning electron microscopy. Varga *et al.* [9] re-validated the species. Our RAPD data and multigene sequence data show that *A. acidus* shows no difference with *A. luchuensis* and *A. kawachii*, and based on priority, the name *A. luchuensis* is proposed as the correct name for these strains.

From our study we can conclude that *A. luchuensis* is the correct name for the fungus used in awamori fermentations and that *A. acidus* and *A. kawachii* are synonyms. Since there has been confusion about the uni- or biseriate feature of the conidial heads of this species, *A. luchuensis* is re-described below.

***Aspergillus luchuensis*** Inui [1], [2].

 =  *Aspergillus perniciosis* Inui [1], [2]

 =  *Aspergillus awamori* Nakazawa pro parte [4]

 =  *Aspergillus aureus* var. *acidus* Nakaz., Simo & A. Watan. [26]

 =  *Aspergillus foetidus* var. *acidus* (Nakaz., Simo & A. Watan.) Thom & Raper [21]

 =  *Aspergillus kawachii* Kitahara & Yoshida [5]

 =  *Aspergillus acidus* Kozak. [27]

 =  *Aspergillus coreanus* Yu et al. [6]

Colony diameters at 7 days, in mm: CYA, 37; DG18, 33; CREA, 29; MEA, 53; MEA37°C, 50.

#### Colony colors and textures

On CYA, sterile mycelium white, conidial areas gray to black, radially sulcate, floccose; reverse cream to brown, radially sulcate. On MEA, obverse, similar to CYA. On CREA, poor growth but with strong acid production.

#### Microscopic characteristics

Conidial heads, radiate; stipes length up to 1.5 mm, width 10–13 µm, walls thick, smooth and hyaline; Vesicles, 20–40 µm wide, nearly spherical; when young uniseriate with irregular formation of metulae, later predominantly biseriate, metulae covering almost entire surface of the vesicle 17.0–26.1 × 4.5–8.1 µm, phialides ampulliform 5.6–8.4 × 3.5–4.9 µm; Conidia, globose, smooth, 3.5–4.5 µm, dark brown.

Type strain: NBRC 4281 =  KACC 46772 =  CBS 205.80 =  IFM 47726 =  RIB 2642, isolated from awamori-koji in Okinawa, Japan, sent by T. Inui to K. Sakaguchi (University of Tokyo).

*Note:* The description above is based on the ex-type strain. As shown in Table S1 and Figure S1 *A. luchuensis* strains show variable morphological characteristic. The colony color is white to gray, or brown to black; stipes have a width 8–30 µm and length up to 1.5 mm; conidial heads are predominantly biseriate, but aberrant uniseriate heads, vesicles are 15–90 µm; metulae 5.0–26.1 µm; phialides are 5.4–12.5 µm; conidia are 3.0–4.5 µm, smooth, finely rough, or rarely rough.

The morphological characteristics are similar with those produced by *A. niger* and *A. tubingensis* in *Aspergillus* section *Nigri,* and it is difficult to differentiate the species based on only morphology. DNA sequences of β-tubulin and calmodulin are useful to differentiate the species from the others, and GenBank JX500062 for β-tubulin and JX500071 for calmodulin could be good molecular markers for the species. Chemically, the extrolites antafumicins [28] and/or “luchuensin” sets *A. luchuensis* apart from all other black *Aspergillus* strains. Antafumicins were first reported from *A. niger* NH-401. We were not able to study this strain, but it is probably *A. luchuensis*.

Inui [1], [2] also described *A. perniciosis* as a contaminant found during awamori-koji fermentation. He distinguished this species by yellow green mycelium with larger conidial heads similar to *A. niger.* No type culture is available, but the description of this taxon resembles *A. coreanus* [6] on the basis of yellow green mycelium. Our molecular analysis shows that *A. coreanus* is a synonym of *A. luchuensis*(Fig. 3). Therefore we consider *A. perniciosis* a further synonym of *A. luchuensis*.

Most isolates of *Aspergillus luchuensis* specifically produce antafumicin and/or a partially characterized metabolite provisionally called “luchuensin”, in addition to asperazine (shared with *A. tubingensis* and *A. vadensis*), they often produce atromentin, funalenone, pyranonigrin A, and occasionally tensidol B [13, 29, 30, Table S2]. None of these extrolites are considered toxic to vertebrates [31]. Furthermore none of these strains produced fumonisin B_2_, B_4_ or B_6_ or any ochratoxins. A total of 52 strains from tea, coffee, and other substrates in addition to industrial strains were analyzed chemically, and none of them produced fumonisins or ochratoxins [13, 32, see also Table S2).

*Aspergillus luchuensis* is the representative fungus of industrial black- and white-koji mold in Japan [11], and it is also the dominant microorganism of Puerh tea in China [13]. The species is also known from nuruk (KACC 41731, 46420), Korean traditional fermentation starter for makgeolli, and meju (KACC 46490), a starter for soybean paste and soy sauce. In the case of meju, out of 54 strains of black *Aspergillus*, 15 strains were *A. luchuensis* (unpublished data). These data suggest that *A. luchuensis* is common in the food fermentation environment and the species play an important role in industry of East Asia.

### Taxonomic Position of *Aspergillus awamori*

The taxonomic position of *A. awamori* Nakazawa proved to be complex. This is mainly caused by the fact that no type of *A. awamori* exists. CBS 557.65 ( =  NRRL 4948), which does not originate from awamori koji and received from Instituto Ozwaldo Cruz, Brazil, was designated as neotype of *A. niger* var. *awamori* by Al-Musallam [14]. The selection of this neotype was based on the fact that Raper and Fennell [7] used this isolate for their description of *A. awamori*. In this study, the neotype of *A. awamori* (CBS 557.65) is located near to *A. niger* (CBS 554.65^T^), but distant from Inui’s *A. luchuensis* (NBRC 4281) (Fig. 3).

Based on the neotype (CBS 557.65), Perrone *et al.* [15] regarded *A. awamori* as a cryptic phylogenetic species in section *Nigri.* In our opinion, the taxonomic position of *A. awamori* is not clear, because no ex-type culture is known while the majority of strains found in culture collections under this name belong to *A. niger* (n = 24), *A. luchuensis* (n = 10), *A. tubingensis* (n = 2) or *A. awamori sensu* Perrone *et al.* (n = 2) (Table 2). According to Yamada *et al.* [11], 14 of 20 strains of *A. awamori* were *A. niger* and six strains were *A. luchuensis*. Out of six of Nakazawa’s strains of *A. awamori*, three strains were the *A. niger* and the other three were *A. luchuensis*. This indicates that Nakazawa’s species concept is not clear and that he dealt with both *A. luchuensis* and *A. niger*. *A. awamori* as described by Nakazawa can be best treated as a doubtful synonym of *A. niger* or *A. luchuensis*.

The strains named as *A. awamori* by Perrone *et al.* [15] based on the incorrect selected neotype (CBS 557.65^NT^) are identical with CBS 139.54, a strain deposited at CBS from *Welwitschia* in Namibia. Figure S2 shows two phylogenetic trees produced from the combined sequence data of two loci (*CaM*, *benA*) of 30 taxa belonging to *A. niger* “aggregate” group. The epitype of *A. welwitschiae* is accommodated in the group of *A. awamori sensu* Perrone *et al.* [15].

Isolates of *A. welwitschiae* have been reported on various substrates as mycotoxin producers or plant pathogens including grapes [15], onions as causative agents of black mold rot and fumonisin contamination [33] and *Welwitschia mirabilis* seeds causing seedling rot (van Diepeningen *et al.* unpublished data). In addition, this species was also identified in various otomycosis cases both in Iran and Hungary [34], [35]. Although the name *A. awamori* was used taxonomically correctly by Perrone *et al.* [15], this currently used name is misleading, as this species is rarely identified in awamori fermentation processes, where mostly *A. niger* and *A. luchuensis* could be isolated [11]. It should also be mentioned that isolates of *A. awamori sensu* Perrone are able to produce ochratoxins and/or fumonisins [15]; [33], making the application of the name of an oriental fermentation process as the basis of the name of this fungus is inadequate.

**Aspergillus welwitschiae** (Bresadola) Hennings apud Wehmer.

 =  *Ustilago welwitschiae* Bres. in Saccardo, Bolm Soc. Broteriana 11∶9–90. 1893 =  *Sterigmatomyces welwitschiae* (Bres.) Henn. in H. Baum Kunene-Zambesi Expedit., p. 168, 1903 =  *Aspergillus welwitschiae* (Bres.) Henn. *apud* Wehmer in Centrbl. Bakteriol. ParasitK. II, 18∶294, 1907

 =  *Aspergillus awamori sensu* Perrone *et al.* [15].

Epitype: CBS 139.54, isolated by H.J. Swart from female inflorescence of *Welwitschia mirabilis* collected in Namibia, stored for 2 years (sample no. 236).

Colonies on CYA, MEA, YES grow similar as *A. niger*. Vesicles 45–85 µm in diameter. Conidia globose, finely to distinctly roughened, brown to dark brown, 3.5–5.5 µm.

Isolates of *Aspergillus niger* and *A. welwitschiae* have overlapping features concerning conidium size, ornamentation, stipe ornamentation, stipe length, and conidium colour. Both species contain mutant isolates that have more brownish conidium colours. *A. welwitschiae* produced conidia which were mostly globose and finely to distinct roughened. The conidial dimensions vary from 3.5–5.5 µm. Vesicles were 45–85 µm in diameter. These morphological characters are identical with the morphological structures in typical *A. niger* strains [30], *A. welwitschiae* has the same ranges of growth rates of *A. niger* on the media CYA at 5, 25 and 37°C, on G25N, CZA, MEA at 25°C in the dark, and also at reduced water activity on CZ20, M40Y at 25°C. It had also a strong acid production on CREA substrate.

Notes: The name *Aspergillus welwitschiae* was first mentioned by Wehmer [36] who reported that this name was proposed by P. Hennings in a written communication. This species has been reported from *Welwitschia mirabilis* by several authors and is mostly called *A. niger* or *A. niger* var. *phoenicis* [37]–[39]. It frequently infects and destroys germinating seeds which has an impact on a *Welwitschia* colony which can sometimes go many years without successfully reproducing.

*Aspergillus welwitschiae* is morphologically indistinguishable from *A. niger* but some fixed nucleotide differences between these two species could be useful for their identification. Sequence positions and differences of *A. welwitschiae* compared to *A. niger* in the four regions sequenced are: calmodulin: 442 (T), 465 (C), 486–87 (CT), 493–494 (TT), 518 (-) 801 (T); β-tubulin: 173339 (T), 173346 (A); - translation elongation factor-1∶665 (G), 669 (A); RNA polymerase II subunit RPB2∶1281 (C), 1449 (C), 1689 (A), 1692 (C), 1719 (C), 1770 (T), 1947 (C), 2103 (T), 2220 (T).

Besides its specific occurrence on *Welwitschia* plants strains of *A. welwitschiae* have been found on grapes, dried fruits, coffee, cocoa, and other sources and it has also a worldwide distribution from all the continents. In particular *A. welwitschiae* resulted to grow very poorly or no growth in the majority of the strains tested on to 2-deoxy-D-glucose substrate, respect to *A. niger* which grows well on this substrate [9].

Most isolates of *A. welwitschiae* produce large amounts of fumonisins and occasionally ochratoxins [32]. The best producer of fumonisin B_2, 4 & 6_ found yet is NRRL 567, a strain identified by us as *A. welwitschiae* and used for citric acid production [32].

## Supporting Information

Figure S1 **Colonies on CYA (left) and conidiophore structure morphology (right) of** ***A. luchuensis*** **and related species.** A-G isolates re-identified as *A. luchuensis* in this study and H and I are *A. niger* and *A. tubingensis*, respectively. A. KACC 46772, B. KACC 46771, C. KACC 46516, D. KACC 45131, E. KACC 41731, F. KACC 46420, G. KACC 46490, H. KACC 45072, I. KACC 46805. Size marker, 10 µm.
(TIF)

Figure S2 **Phylogenetic trees produced from the combined sequence data of two loci (** ***CaM*** **,** ***benA*** **) of 30 taxa, including** ***A. welwitschiae*** **and** ***A. awamori sensu*** **Perrone** ***et al.*** [**15**] **belonging to** ***A. niger*** **“aggregate” group.** Numbers above branches are bootstrap values**.** Only values above 70% are indicated. The evolutionary history was inferred using the Neighbor-Joining (A) and the Maximum Parsimony method (B).
(TIF)

Table S1 **Morphological characteristics of** ***A. luchuensis*** **and related species.**
(DOCX)

Table S2 **Extrolites found in strains identified as** ***Aspergillus luchuensis*** **(formerly** ***A. acidus*** **).**
(DOCX)
