# Supplementary figures and images for: ASPERGILLUS LUCHUENSIS , AN INDUSTRIALLY IMPORTANT BLACK ASPERGILLUS IN EAST ASIA
Source: PLoS One. 2013 May 28;8(5):e63769. doi: 10.1371/journal.pone.0063769 (PMC3665839; doi:10.1371/journal.pone.0063769)

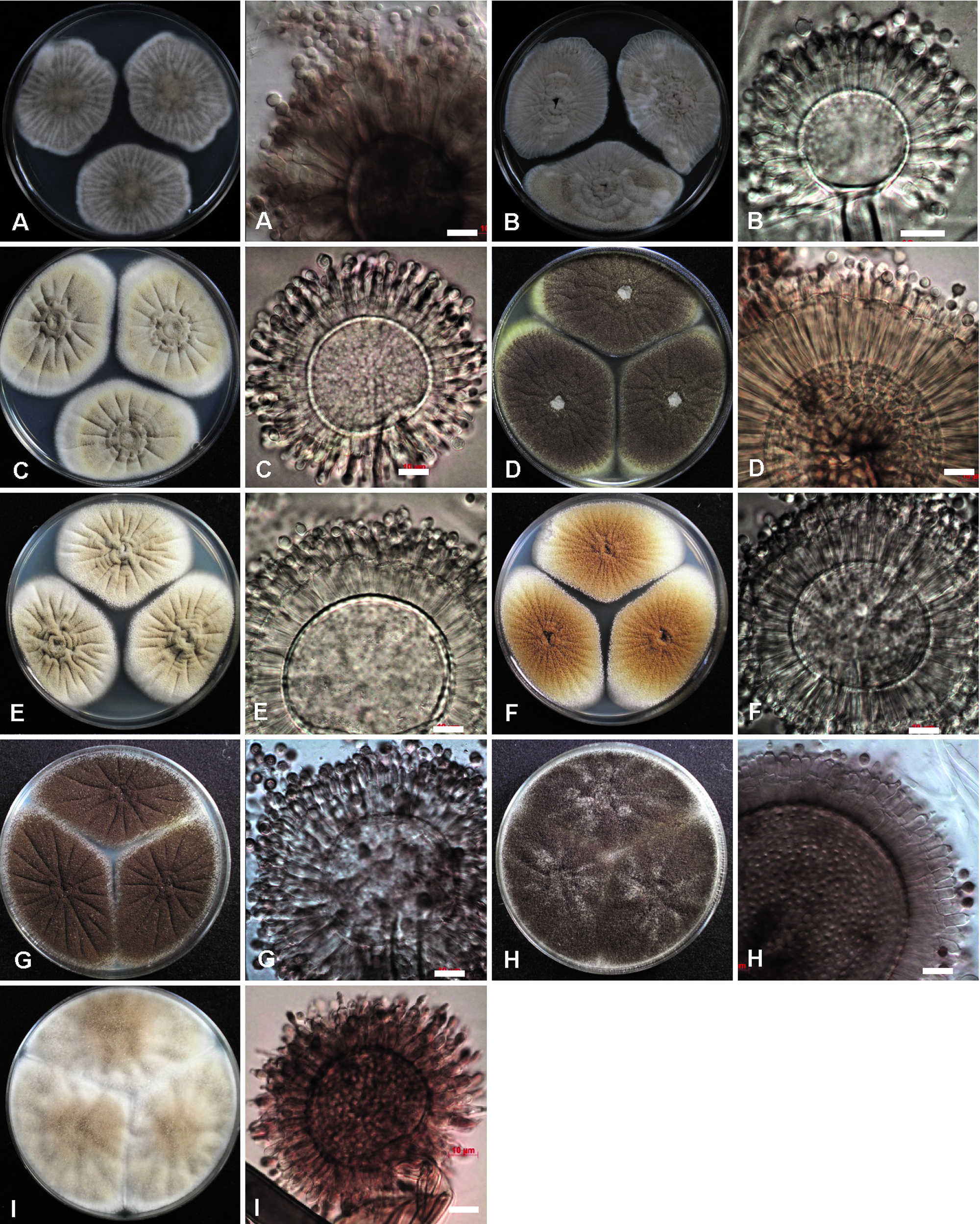

Supplement: Figure S1 — Colonies on CYA (left) and conidiophore structure morphology (right) of A. luchuensis and related species. A-G isolates re-identified as A. luchuensis in this study and H and I are A. niger and A. tubingensis, respectively. A. KACC 46772, B. KACC 46771, C. KACC 46516, D. KACC 45131, E. KACC 41731, F. KACC 46420, G. KACC 46490, H. KACC 45072, I. KACC 46805. Size marker, 10 µm. (TIF) [file pone.0063769.s001.tif]

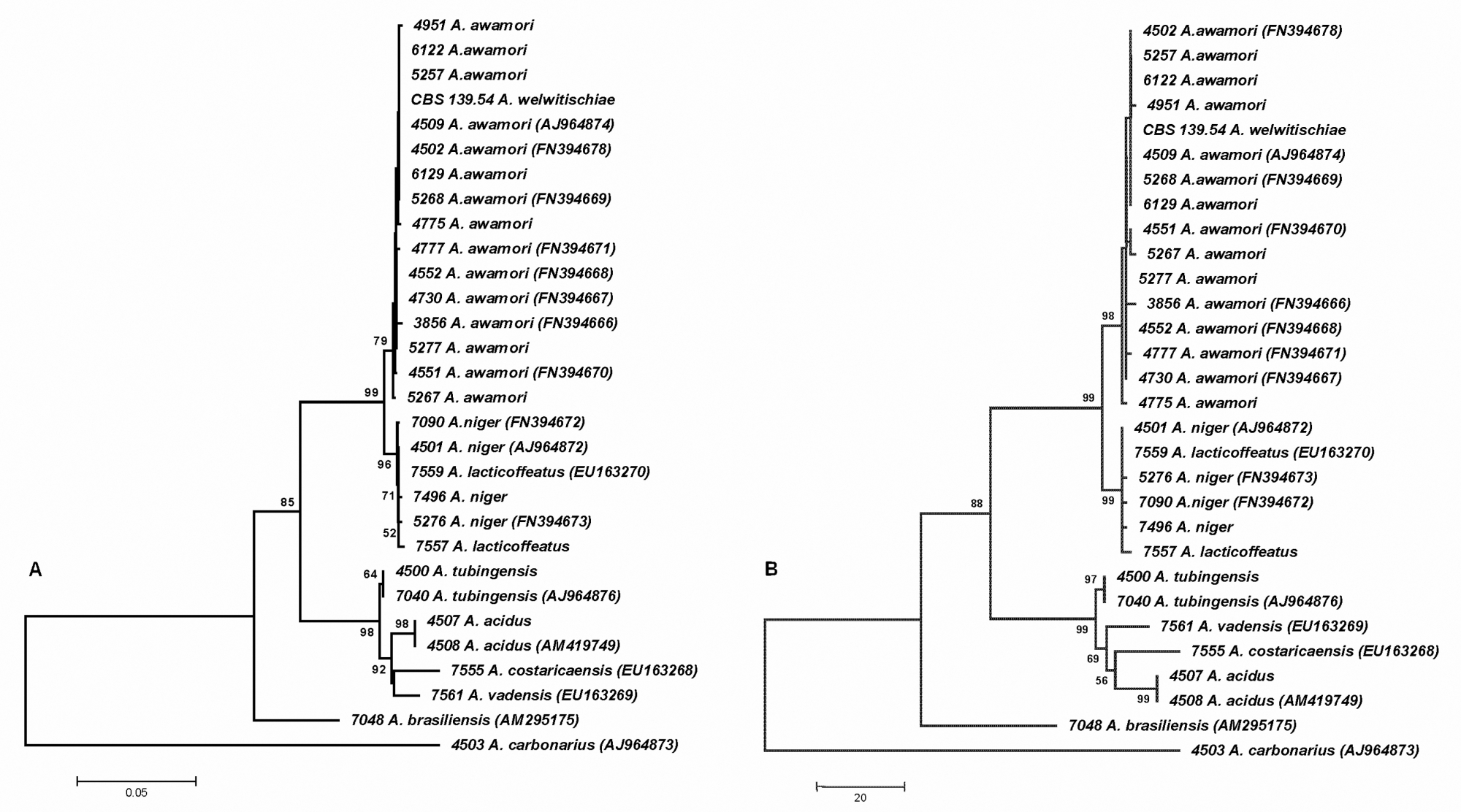

Supplement: Figure S2 — Phylogenetic trees produced from the combined sequence data of two loci ( CaM , benA ) of 30 taxa, including A. welwitschiae and A. awamori sensu Perrone et al. [15] belonging to A. niger “aggregate” group. Numbers above branches are bootstrap values. Only values above 70% are indicated. The evolutionary history was inferred using the Neighbor-Joining (A) and the Maximum Parsimony method (B). (TIF) [file pone.0063769.s002.tif]
