# Supplementary material for: ASPERGILLUS LUCHUENSIS , AN INDUSTRIALLY IMPORTANT BLACK ASPERGILLUS IN EAST ASIA
Source: PLoS One. 2013 May 28;8(5):e63769. doi: 10.1371/journal.pone.0063769 (PMC3665839; doi:10.1371/journal.pone.0063769)
Supplement: Table S1 — Morphological characteristics of A. luchuensis and related species. (DOCX) [file pone.0063769.s003.docx]

Supplementary Table 1. Morphological characteristics of *A. luchuensis* and related species.

| KACC No. | Scientific name  (After this study) | Colony color | Conidiophores (width, length, µm) | Vesicles (µm) | Metulae (µm) | Phialides (µm) | Conidia (shape, size-µm, ornamentation) |
| --- | --- | --- | --- | --- | --- | --- | --- |
| 46772 | *Aspergillus luchuensis* | gray to brown black | 10–13, up to 1500 | 20–40, gl*. | 17.0–26.1 | 5.6–8.4 | gl*., 3.5–4.5, sm*. |
| 46771 | *A. luchuensis* | white to gray | 8–12, up to 500 | 18–36, gl. | 5.0–8.2 | 5.4–9.2 | gl., 3.0–4.0, sm. |
| 46516 | *A. luchuensis* | gray | 10–15, up to 600 | 15–30, gl. | 9.1–13.6 | 7.2–9.9 | gl., 3.3–4.3, sm. |
| 45131 | *A. luchuensis* | dark brown | 15–30, up to 850 | 50–90, gl | 11.1–19.3 | 8.3–11.1 | gl., 3.5–4.5, sm, fr*. |
| 45132 | *Aspergillus* sp*.* | black | 15–20, up to 1500 | 40–70, gl | 22–29 | 8–10 | gl, 3.0–4.0, rf* |
| 45133 | *Aspergillus* sp. | black | 10–15, up to 1000 | 45–63, gl | 20–35 | 8–12 | gl, 3.0–4.0, rf |
| 41731 | *A. luchuensis* | light brown | 12–18, up to 800 | 27–60, gl | 8.1–13.4 | 8.6–12.5 | gl, 3.0–4.0, sm |
| 46420 | *A. luchuensis* | brown | 13–18, up to 600 | 38–63, gl | 12.6–24.4 | 8.5–11.5 | gl., 3.0–4.5, sm, fr. |
| 46490 | *A. luchuensis* | brown black to black | 13–20, up to 600 | 40–65, gl | 16–20 | 7–12 | gl., 3.0–4.0, rf |
| 45072 | *A. niger* | black | 15–23, up to 2000 | 60–75, gl | 17–27 | 8–10 | gl., 3.0–4.5, rf |
| 46805 | *A. tubingensis* | brown to brown black | 14–35, up to 5000 | 25–75, gl | 10.8–32.3 (80) | 8–12 | gl., 3.2–4.2, rf |

* gl., globose; sm., smooth; fr., finely rough; rf., rough.
