# Supplementary material for: ASPERGILLUS LUCHUENSIS , AN INDUSTRIALLY IMPORTANT BLACK ASPERGILLUS IN EAST ASIA
Source: PLoS One. 2013 May 28;8(5):e63769. doi: 10.1371/journal.pone.0063769 (PMC3665839; doi:10.1371/journal.pone.0063769)
Supplement: Table S2 — Extrolites found in strains identified as Aspergillus luchuensis (formerly A. acidus ). (DOCX) [file pone.0063769.s004.docx]

Supplementary Table 2. Extrolites found in strains identified as *Aspergillus luchuensis* (formerly *A. acidus*)

| CBS 114.52 = WB 4842 = IBT 4935 | antafumicin, asperazine, funalenone, naphtho-γ-pyrones, pyranonigrin B (fumonsin and OTA absent) |
| --- | --- |
| CBS 117.32 = IBT 3252 | antafumicin, asperazine (fumonsin and OTA absent) |
| CBS 565.65 = IFO 4123 = WB 4797 = ATCC 16884 = IMI 175963 = IBT 3644 | antafumicin, asperazine, atromentin, funalenone, naphtho-γ-pyrones, pyranonigrin A, tensidol B, (“luchuensin”) (fumonsin and OTA absent) |
| CBS 105.47 = IBT 3412 | antafumicin, asperazine, atromentin, funalenone, naphtho-γ-pyrones, pyranonigrin A (fumonsin and OTA absent) |
| CBS 107.47 | (fumonsin and OTA absent) |
| CBS 564.65 = IBT 3410 = IMI 104688 = ATCC 16874 = IFO 4121 = WB 4796 = IBT 4570 = IBT 4602 | antafumicin, asperazine, atromentin, pyranonigrin A, (“luchuensin”) (fumonsin and OTA absent) |
| CBS 115.52 = ATCC 11358 = IBT 23470 | antafumicin, asperazine, funalenone, naphtho-γ-pyrones, pyranonigrin A (“luchuensin”) (fumonsin and OTA absent) (fumonsin and OTA absent) |
| CBS 103.14 = IFO 4338 = QM 9706 = IBT 4973 | asperazine, funalenone, asperazine, naphtho-γ-pyrones |
| CBS 111.34 = IBT 4354 = ?IFO 4033 | asperazine, atromentin, naphtho-γ-pyrones, pyranonigrin A |
| CBS 118.48 = IBT 4952 | asperazine, atromentin, naphtho-γ-pyrones, pyranonigrin A |
| CBS 122.48 = QM 9768 = IBT 30189 | antafumicins, asperazine, funalenone, naphtho-γ-pyrones |
| CBS 124.49 = IBT 28077 | antafumicin, asperazine, atromentin, funalenone, naphtho-γ-pyrones,pyranonigrin A |
| CBS 119.52 | (fumonsin and OTA absent) |
| CBS 126.52 | (fumonsin and OTA absent) |
| CBS 619.78 = NRRL 4794 = IBT 30188 | asperazine, funalenone, naphtho-γ-pyrones, pyranonigrin B |
| CBS 119384 = KACC 41731 = KCTC 18075P = KCCM 80006 = IBT 27923 | asperazine, pyranonigrin A (fumonsin and OTA absent) |
| KACC 41733 = IBT 27924 | asperazine, pyranonigrin A, (“luchuensin”) (*A. coreanus*) (fumonsin and OTA absent) |
| CBS 128.52 = QM 8183 = WB 4750 = NRRL 4750 = IBT 29904 | thin, only a tetracyclic compound (fumonsin and OTA absent) |
| NRRL 356 = QM 334 = ATCC 10061 = IMI 015953 = Thom 4291.3 = LSHB Ac88= IBT 15244 | asperazine, funalenone, naphtho-γ-pyrones, pyranonigrin A |
| NRRL 2322 = NRRL 2354 = IBT 28862 = ATCC 10577 = IMI 027809 = NCTC 7193 = QM 6906 = DSMZ 823 = IBT 29956 | asperazine, atromentin, funalenone, naphtho-γ-pyrones, pyranonigrin A, tensidol B (fumonsin and OTA absent) |
| CBS 553.65 = WB 5121 = IMI 235599 = ATCC 16880 = IBT 28093 | antafumicins, asperazine, naphtho-γ-pyrones (fumonsin and OTA absent) |
| IBT 4935 | (fumonsin and OTA absent) |
| IBT 16906 | (fumonsin and OTA absent) |
| IBT 21580 | antafumicin, asperazine, funalenone, naphtho-γ-pyrones, pyranonigrin A |
| IBT 20295 | antafumicin, asperazine, funalenone, naphtho-γ-pyrones, pyranonigrin A |
| IBT 20290 | antafumicin, asperazine, funalenone, naphtho-γ-pyrones, pyranonigrin A, (“luchuensin”) |
| IBT 20297 | asperazine, funalenone, naphtho-γ-pyrones, pyranonigrin A, (“luchuensin”) |
| IBT 20305 | asperazine, funalenone, naphtho-γ-pyrones, pyranonigrin A, (“luchuensin”) |
| IBT 20370 | asperazine, funalenone, naphtho-γ-pyrones, pyranonigrin A, (“luchuensin”) |
| IBT 20376 | asperazine, funalenone, naphtho-γ-pyrones, pyranonigrin A, (“luchuensin”) |
| IBT 21790 | asperazine, funalenone, naphtho-γ-pyrones, pyranonigrin A, (“luchuensin”) |
| IBT 23772 | asperazine, funalenone, naphtho-γ-pyrones, pyranonigrin A, (“luchuensin”) |
| IBT 24822 | antafumicin, asperazine, funalenone, naphtho-γ-pyrones, pyranonigrin A, (“luchuensin”) |
| IBT 24831 | antafumicin, asperazine, funalenone, naphtho-γ-pyrones, nigragillin, pyranonigrin A, (“luchuensin”) |
| IBT 24800 | antafumicin, asperazine, funalenone, naphtho-γ-pyrones, pyranonigrin A (“luchuensin”) |
| IBT 24799 | antafumicin, asperazine, funalenone, naphtho-γ-pyrones, nigragillin, pyranonigrin A, (“luchuensin”) |
| IBT 24798 | antafumicin, asperazine, funalenone, naphtho-γ-pyrones, nigragillin, pyranonigrin A, (“luchuensin”) |
| IBT 24830 | asperazine, funalenone, naphtho-γ-pyrones, nigragillin, (“luchuensin”) |
| IBT 24825 | antafumicin, asperazine, funalenone, naphtho-γ-pyrones, pyranonigrin A, (“luchuensin”) (fumonsin and OTA absent) |
| IBT 24803 | asperazine, funalenone, naphtho-γ-pyrones, nigragillin, pyranonigrin A, (“luchuensin”) |
| IBT 24826 | antafumicin, asperazine, funalenone, naphtho-γ-pyrones, pyranonigrin A, (“luchuensin”) |
| IBT 24802 | asperazine, atromentin, funalenone, naphtho-γ-pyrones, pyranonigrin A, (“luchuensin”) |
| IBT 24801 | asperazine, atromentin, funalenone, naphtho-γ-pyrones, pyranonigrin A, (“luchuensin”) |
| IBT 24821 | asperazine, funalenone, naphtho-γ-pyrones, pyranonigrin A, (“luchuensin”) |
| IBT 28087 | asperazine, funalenone, atromentin, naphtho-γ-pyrones, pyranonigrin A, (“luchuensin”) |
| IBT 29212 | asperazine, funalenone, naphtho-γ-pyrones, pyranonigrin A, (“luchuensin”) |
| IBT 29214 | (fumonsin and OTA absent) |
| IBT 29217 | antafumicins, asperazine, atromentin, funalenone, naphtho-γ-pyrones,(“luchuensin”) (fumonsin and OTA absent) |
| IBT 29220 | antafumicins, asperazine, atromentin, funalenone, naphtho-γ-pyrones,(“luchuensin”) (fumonsin and OTA absent) |
| IBT 29221 | antafumicins, asperazine, atromentin, funalenone, naphtho-γ-pyrones,(“luchuensin”) (fumonsin and OTA absent) |
| IBT 29199 | asperazine, atromentin, funalenone, naphtho-γ-pyrones,(“luchuensin”) |
| IBT 29186 | asperazine, atromentin, funalenone, naphtho-γ-pyrones,(“luchuensin”) (fumonsin and OTA absent) |
| Coffee Thailand, 1B9 = ? | antafumicins, asperazine, atromentin, naphtho-γ-pyrones, pyranonigrin A, (“luchuensin”) |
| Coffee Thailand, 8H7 | antafumicins, asperazine, atromentin, funalenone, naphtho-γ-pyrones, pyranonigrin A, (“luchuensin”) |
| Coffee Thailand 9A2 | antafumicins, asperazine, atromentin, funalenone, naphtho-γ-pyrones, pyranonigrin A, (“luchuensin”) |
| Coffee Thailand 9F1 | antafumicins, asperazine, atromentin, funalenone, naphtho-γ-pyrones, pyranonigrin A, (“luchuensin”) |
| Coffee Thailand 1G2 | antafumicins, asperazine, atromentin, funalenone, naphtho-γ-pyrones, pyranonigrin A, (“luchuensin”) |
| Coffee Thailand 1F2 | asperazine, atromentin, funalenone, naphtho-γ-pyrones, pyranonigrin A, (“luchuensin”) |
| Coffee Thailand 2C5 | asperazine, atromentin, funalenone, naphtho-γ-pyrones, pyranonigrin A, (“luchuensin”) |
| Coffee Thailand 2C7 | asperazine, atromentin, funalenone, naphtho-γ-pyrones, pyranonigrin A, (“luchuensin”) |
| Coffee Thailand 2C8 | asperazine, atromentin, funalenone, naphtho-γ-pyrones, pyranonigrin A, (“luchuensin”) |
| Coffee Thailand 6D9 | asperazine, atromentin, funalenone, naphtho-γ-pyrones, pyranonigrin A, (“luchuensin”) |
| Coffee Thailand 6B4 | antafumicin, asperazine, atromentin, funalenone, naphtho-γ-pyrones, pyranonigrin A, (“luchuensin”) |
| Coffee Thailand 6H4 | antafumicin, asperazine, atromentin, funalenone, naphtho-γ-pyrones, pyranonigrin A, (“luchuensin”) |
| Coffee Thailand 8D8 | antafumicin, asperazine, atromentin, funalenone, naphtho-γ-pyrones, pyranonigrin A, (“luchuensin”) |
| Coffee Thailand 8F4 | antafumicin, asperazine, atromentin, funalenone, naphtho-γ-pyrones, pyranonigrin A, (“luchuensin”) |
| Coffee Thailand 8F6 | antafumicin, asperazine, atromentin, funalenone, naphtho-γ-pyrones, pyranonigrin A, (“luchuensin”) |

Further data on tea strains in Mogensen et al. (2009), fumonsin and OTA absent. Of 42 strains tested none produced fumonisins or ochratoxins [32]. Of 47 strains tested from black tea, none produced fumonisins or ochratoxins [13].
